# Comparison of galactomannan lateral flow assay and enzyme immunoassay to identify *Aspergillus spp*. in bronchoalveolar lavage fluid

**DOI:** 10.1016/j.bjid.2024.103838

**Published:** 2024-07-14

**Authors:** Sarah Craveiro Martins, Cibele Aparecida Tararam, Larissa Ortolan Levy, Teppei Arai, Akira Watanabe, Maria Luiza Moretti, Plínio Trabasso

**Affiliations:** a Universidade Estadual de Campinas (Unicamp) – Hospital das Clínicas, Faculdade de Ciências Médicas, Laboratório de Epidemiologia Molecular e Doenças Infecciosas (LEMDI), Cidade Universitária, Campinas, SP, Brazil; b Chiba University, Division of Clinical Research, Medical Mycology Research Center (MMRC), Inohana, Chuo-ku, Chiba-shi, Chiba, Japan

**Keywords:** Diagnosis, Aspergillosis, Galactomannan, Lateral flow assay, Enzyme immunoassay

## Abstract

*Aspergillus* species can colonize and infect immunocompetent and immunocompromised hosts. Conventional fungal identification depends on microscopic analysis and microorganism medium growth. Other diagnostic methods, non-growth dependent, to invasive fungal infections, are the biomarkers that detect circulating polysaccharides, for example, 1-3-β-d-Glucan and galactomannan. Both are polysaccharides present on the external layer of fungi cell wall and can be detected in clinical samples during the growth of the fungus in the patient. This study aimed to compare the galactomannan detection of Lateral Flow Assay and Enzyme Immunoassay methods in Bronchoalveolar Lavage Fluid. The galactomannan antigen in Bronchoalveolar Lavage Fluid was measured using Enzyme Immunoassay according to the manufacturer's instructions (PLATELIA ASPERGILLUS™ BioRad) and, using a Lateral Flow Assay according to the manufacturer's instructions (Galactomannan LFA IMMY©). The 71 samples were Bronchoalveolar Lavage Fluid of patients hospitalized at Unicamp Clinical Hospital between 2019 and 2021; of these samples 12/71 (16.9 %) resulted in positive Galactomannan-Lateral Flow Assay. In contrast, Galactomannan-Enzyme Immunoassay resulted as positive in 9/71 (12.6 %) samples, a difference that showed not significant statistically (p-value = 0.36) Comparing both assays’ results identified 8 divergences between them, about 11 % of the total sample. The Sensitivity (73.3 %), Specificity (92.35 %), Positive Predictive Value (62.85 %) and Negative Predictive Value (95.15 %) of Lateral Flow Assay were calculated using the Galactomannan Enzyme Immunoassay as standard. The Lateral Flow Assay demonstrated good results when compared with the Enzyme Immunoassay.

## Introduction

Fungi are eukaryotic organisms found in the most diverse habitats.^1^ Among the higher clinical relevance pathogenic fungi are those of the genus *Aspergillus*, a filamentous fungi of environmental origin, responsible for high mortality levels.^2^ The airborne conidia are the infective form, and the pathogen exposure occurs after the fungi are consumed or inhaled.^3^

The clinically relevant fungi are mainly distributed between the sections Fumigati, Flavi, Nigri, Nidulantes, Usti, and Terrei^4^^,^^5^ The main aspergillosis-causing species is *A. fumigatus*,^6^ followed by *A. flavus*,^7^ *A. niger*, and *A. terreus*.^8^, ^9^, ^10^ *Aspergillus* species can infect and colonize immunocompetent and immunocompromised hosts.^11^

Conventional fungal identification depends on microscopic analysis and microorganism medium growth.^12^^,^^13^ The definitive identification consists of the clinical sample cultivation and agent growth in culture, which is still considered a gold standard for laboratory diagnosis of infections.^14^

Other diagnostic methods, non-growth dependent on invasive fungal infections, are the biomarkers that detect circulating polysaccharides, for example, 1-3-β-d-Glucan and Galactomannan.^15^ Both are polysaccharides present on the external layer of the fungi cell wall, which can be detected in clinical samples during the growth of the fungus in the patient.^16^^,^^17^

While galactomannan is usually detected with an Enzyme Immunoassay (EIA), the EIA is not broadly available in low and middle-income countries (LMICs), and turnaround time may be a limitation.^18^ The *Aspergillus*-specific Galactomannan Lateral Flow Assay (GM-LFA) is a simple and rapid test that may overcome some of those limitations, as it only requires rudimentary laboratory facilities and is featured by rapid turnaround time.^19^^,^^20^

In this context, new methodologies are promising tools that reduce the identification time and allow a more precise and reliable diagnostic. This study aimed to compare the galactomannan detection of LFA and EIA methods in Bronchoalveolar Lavage Fluid (BALF).

## Material and methods

### Study design

A retrospective analysis was performed in BALF samples to compare the galactomannan detection of LFA and EIA methods. This study was approved by the Research Ethics Committee – CEP from the State University of Campinas, with the number of Certificate of Presentation of Ethical Review (CAAE): 79558124.8.0000.5404.

### BALF samples

BALF samples were collected between 2019 and 2021 from patients admitted at the Hospital das Clínicas da Universidade de Campinas (Unicamp) with clinical suspicion of Invasive Pulmonary Aspergillosis (IPA). After the measurement of GM by EIA, the samples were stored at −80 °C in the Laboratory of Molecular Epidemiology and Infectious Diseases, School of Medical Sciences, Unicamp.

### Galactomannan enzyme immunoassay

The galactomannan in BALF was measured by the EIA technique, according to the manufacturer's instructions (Platelia *Aspergillus*™ BioRad, Hercules, California, U.S.A.). All BALF samples were tested fresh immediately after collection. The tests were considered positive if the cutoff value was ≥0.50 Optical Density Index (ODI).

### Galactomannan lateral flow assay

The galactomannan antigen in BALF was measured using a Lateral Flow Assay according to the manufacturer's instructions (Soña Aspergillus GM Lateral Flow Assay – IMMY©, Norman, Oklahoma, USA). We used the BALF samples previously stored at −80 °C. The Sona LFA cube reader (IMMY Diagnostics) was used when reading each LFA to remove subjectivity, confirm validity, and provide a GM index. The tests were considered positive if the cutoff value was ≥0.50 ODI.

### Data analysis

The diagnostic performance of GM assay in BALF (GM-Cutoff ≥0.5 ODI) was evaluated by calculating sensitivity, specificity, positive predictive value, negative predictive value, and accuracy, between GM-EIA and GM-LFA, using the GM-EIA as standard.

## Results

A total of 71 samples, 37 (52.1 %) collected in 2019, 21 (29.6 %) in 2020, and 13 (18.3 %) in 2021 were analyzed. The GM-LFA resulted in positive in 12/71 (16.9 %) BALF samples. In contrast, GM-EIA resulted in positive in 9/71 (12.6 %) samples, although the difference is not statistically significant (p-value = 0.36).

The results of both tests were primarily consistent, except for eight samples (11.3 %), in which five were positive by LFA but negative by GM-EIA, and three which were negative by LFA but positive by GM-EIA (Table 1). Of 71 BALF samples, 6 (8.45 %) showed positivity in both techniques GM-EIA and GM-LFA.

**Table 1:** The divergences between Galactomannan Lateral Flow Assay (GM-LFA) and Galactomannan EIA Immunoassay (GM-EIA) in Bronchoalveolar Lavage Fluid (BALF) (*n* = 8).

Sample	Year	GM-LFA	Value	GM-EIA	Value
5085	2019	Positive	0.55	Negative	0.18
5088	2019	Positive	0.63	Negative	0.14
5569	2019	Positive	0.99	Negative	0.06
6139	2020	Positive	1.21	Negative	0.14
6788	2021	Positive	0.62	Negative	0.41
5434	2019	Negative	0.05	Positive	0.87
5610	2019	Negative	0.26	Positive	5.27
6912	2020	Negative	0.49	Positive	0.83

GM, Galactomannan; LFA, Lateral Flow Assay; EIA, Enzyme Immunoassay.

As shown in Table 1, among the eight samples where the results of the assays were divergent, five were collected in 2019 (62.5 %). Considering that the GM-EIA assays were performed with fresh samples and the GM-LFA with frozen samples, this factor can be considered a limitation of the analysis.

Table 2 shows a comparative between the results of both assays. The sensitivity (73.3 %), specificity (92.35 %), positive and negative predictive values (62.9 %/95.2 %), and accuracy (90.1 %) for galactomannan lateral flow assay were calculated using the galactomannan EIA as the golden standard in BALF (GM-Cutoff ≥0.5 ODI). The sensitivity and specificity of GM-LFA were 73.3 and 92.35, respectively.

**Table 2:** Comparison between positive and negative results for GM-LFA and GM-EIA.

GM LFA	GM EIA		Total
	Positive	Negative	
Positive	7	5	12
Negative	3	56	59
Total	10	61	71

GM, Galactomannan; ODI, Cutoff Optical Density Index; LFA, Lateral Flow Assay; PPV, Positive Predictive Value; NPV, Negative Predictive Value.

When changing the GM-Cutoff to ≥1.0 ODI, indicated by some authors when analyzing BALF samples,^21^^,^^22^ different results were found. With the same total of 71 samples, GM-LFA resulted in positive in 5/71 (7.04 %) BALF samples, while GM-EIA resulted in positive in 7/71 (9.85 %) samples.

Comparing the results of both tests, four samples (5.6 %), in which one were positive by LFA but negative by GM-EIA, and three were negative by LFA but positive by GM-EIA (Table 3). Of 71 BALF samples, 4 (5.6 %) showed positivity in both techniques GM-EIA and GM-LFA.

**Table 3:** The divergences between Galactomannan Lateral Flow Assay (GM-LFA) and Galactomannan EIA Immunoassay (GM-EIA) in Bronchoalveolar Lavage Fluid (BALF) using GM-Cutoff ≥ 1.0 ODI (*n* = 4).

Sample	Year	GM-LFA	Value	GM-EIA	Value
5178	2019	Negative	0.54	Positive	2.12
5610	2019	Negative	0.26	Positive	5.1
6139	2020	Positive	1.21	Negative	0.13
6894	2021	Negative	0.59	Positive	1.08

GM, Galactomannan; LFA, Lateral Flow Assay; EIA, Enzyme Immunoassay.

Table 4 shows a comparative between the results of both assays. The sensitivity (57.14 %), specificity (95.45 %), positive and negative predictive values (80 %/95.45 %), and accuracy (94.37 %) for galactomannan lateral flow assay were calculated using the galactomannan EIA as the golden standard in BALF (GM-Cutoff ≥1.0 ODI).

**Table 4:** Comparison between positive and negative results for GM-LFA and GM-EIA using GM-Cutoff ≥1.0 ODI.

GM LFA	GM EIA		Total
	Positive	Negative	
Positive	4	1	5
Negative	3	63	66
Total	7	64	71

GM, Galactomannan; ODI, Cutoff Optical Density Index; LFA, Lateral Flow Assay; PPV, Positive Predictive Value; NPV, Negative Predictive Value.

## Discussion

In this study, we investigated the performance of a new *Aspergillus* GM-LFA for detecting the GM antigen in BALF in hospitalized populations, compared to the GM-EIA.

We studied 71 samples of BALF from patients with aspergillosis suspicion hospitalized at the Clinical Hospital of Unicamp at Campinas, São Paulo – Brazil. Conventional mycological diagnostics may have insufficient sensitivities to diagnose Invasive Aspergillosis (IA). Due to the imperfect sensitivity of conventional diagnostic tests,^23^^,^^24^ serological and molecular methods have become a cornerstone in diagnosing IA.^25^ Particularly GM testing from BALF and serum is now widely used for diagnosis and treatment stratification in IA.^26^^,^^19^

The development of LFA is seen as an important innovation in terms of mycological sciences.^27^ In particular, the practicality of the test, its early results, and the ability to work in different body fluids other than serum are among its critical advantages.

Sensitivities and specificities of the GM-LFA found in our study were comparable with previous studies. In this present study, the *Aspergillus* LFA test has shown to be a reliable alternative with results that strongly correlate with GM-EIA testing, with high sensitivity (73.3 %) and specificity (92.3 %) in BAL fluid. Jenks et al., using a cutoff of > 0.5, showed a sensitivity of 89 % and a specificity of 44 % in a total of 296 BALF samples.^20^ In the study of Ghazanfari et al., using a GM index ≥ 1.0, found a similar sensitivity (60.6 %) and specificity (88.9 %) compared to the present; however, they included 33 BALF samples and used a higher cut-off value,^19^ despite that, in this study, when changing the cut-off value, the sensitivity decreased (73.3 % to 57.14 %) and the specificity increased (92.3 % to 95.45 %), also, the positive and negative predictive values and accuracy increased compared to cut-off of GM index ≥ 0.5.

Conversely, the sensitivity and specificity found by Jani et al., using the GM-EIA as the gold standard, was higher than this study, 100 % and 93 %, respectively, in total, included 90 BALF samples from a cancer population.^28^

The study of Jani and collaborators also obtained samples with divergent results; as of 90 samples, 6 (6.7 %) presented a different result between LFA and EIA assays, where all samples showed a negative EIA result and LFA positive. In our study, 8/71 (11.3 %) of samples were divergent, and 5/71 (7.0 %) were positive for LFA and negative for EIA, similar to previous studies.

Limitations of our study include the single center, the retrospective design, and the fact that the BALF GM results were not clinically evaluated with criteria for invasive aspergillosis or performed other diagnostic methods, such as culture, microscopy, and molecular biology. Also, the GM-LFA was performed using BALF previously stored in an ultra-freezer, which may have affected the results.

## Conclusion

The diagnosis of the etiologic agent of the fungal infection is the key point for the early determination of the infection and adequate therapy, the *Aspergillus* Galactomannan LFA with the reader demonstrated good results when compared with the GM-EIA. It can be a resourceful tool for IA diagnosis in BALF samples, the GM-LFA showed to be a rapid test with a great cost benefit.

It is recommended to combine the methods in many studies, with a larger study population, to provide a better inference about this new test and provide a superior early diagnosis for IA.

## Funding

This work was supported by the Japan International Cooperation Agency (JICA) with the collaboration of the Medical Mycology Research Center (MMRC) of Chiba University.

## Conflicts of interest

The authors declare no have conflicts of interest.
